# Supplementary material for: Evaluating the Genome-Based Average Nucleotide Identity Calculation for Identification of Twelve Yeast Species
Source: J Fungi (Basel). 2024 Sep 11;10(9):646. doi: 10.3390/jof10090646 (PMC11433037; doi:10.3390/jof10090646)
Supplement: Supplementary file 1 [file jof-10-00646-s001.zip › Supplementary Figures.pdf]

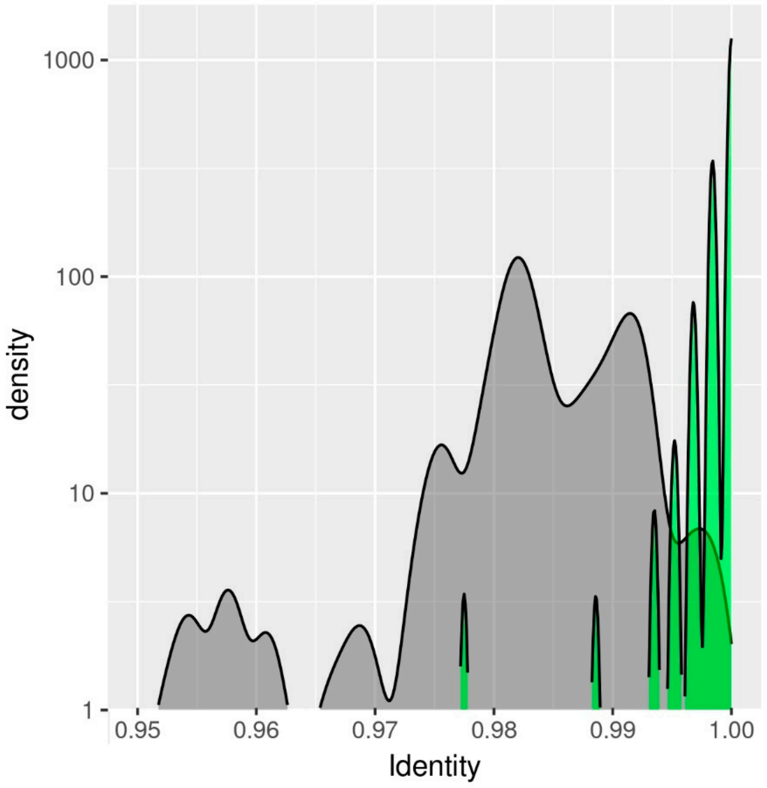

Figure S1. Density curves of D1/D2 region limited to identity values between 95% and 100%. Y axis is in logarithmic scale. Green curve depicts values from conspecific assemblies and grey curve corresponds to ID values from different species assemblies.

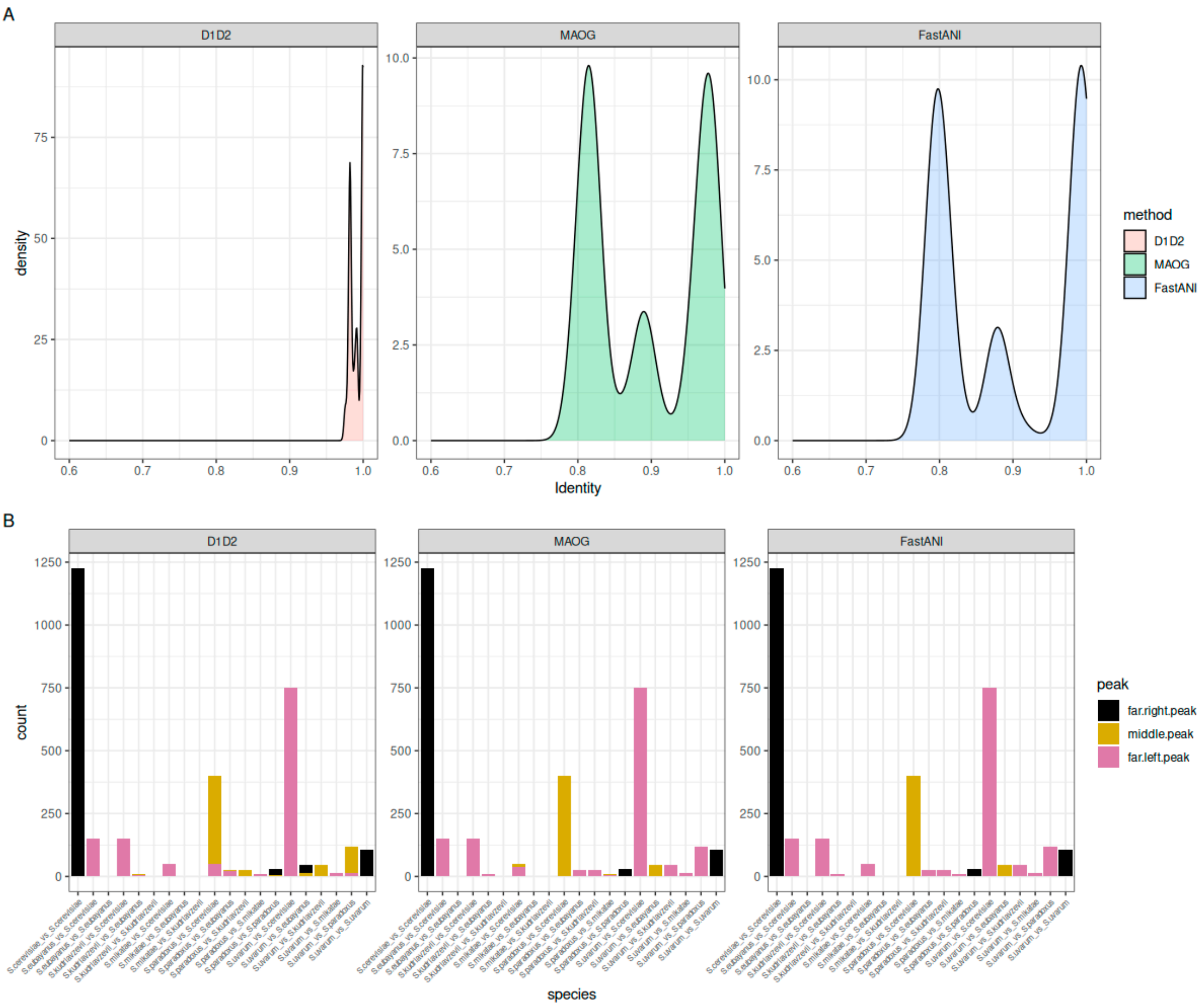

Figure S2. A) Density estimation of the pairwise identity values obtained for Saccharomyces assemblies in the original dataset, calculated with D1/D2, MAOG, and FastANI methods. B) Histograms of pairwise identity values among *Saccharomyces* assemblies according to the species they belong to. Color indicates the peak at which an identity value was found in the density plot in panel A. This classification was made taking into account the minimum nodes calculated for each method (D1/D2: 0.987 and 0.995; MAOG: 0.858 and 0.927; FastANI: 0.845 and 0.938).

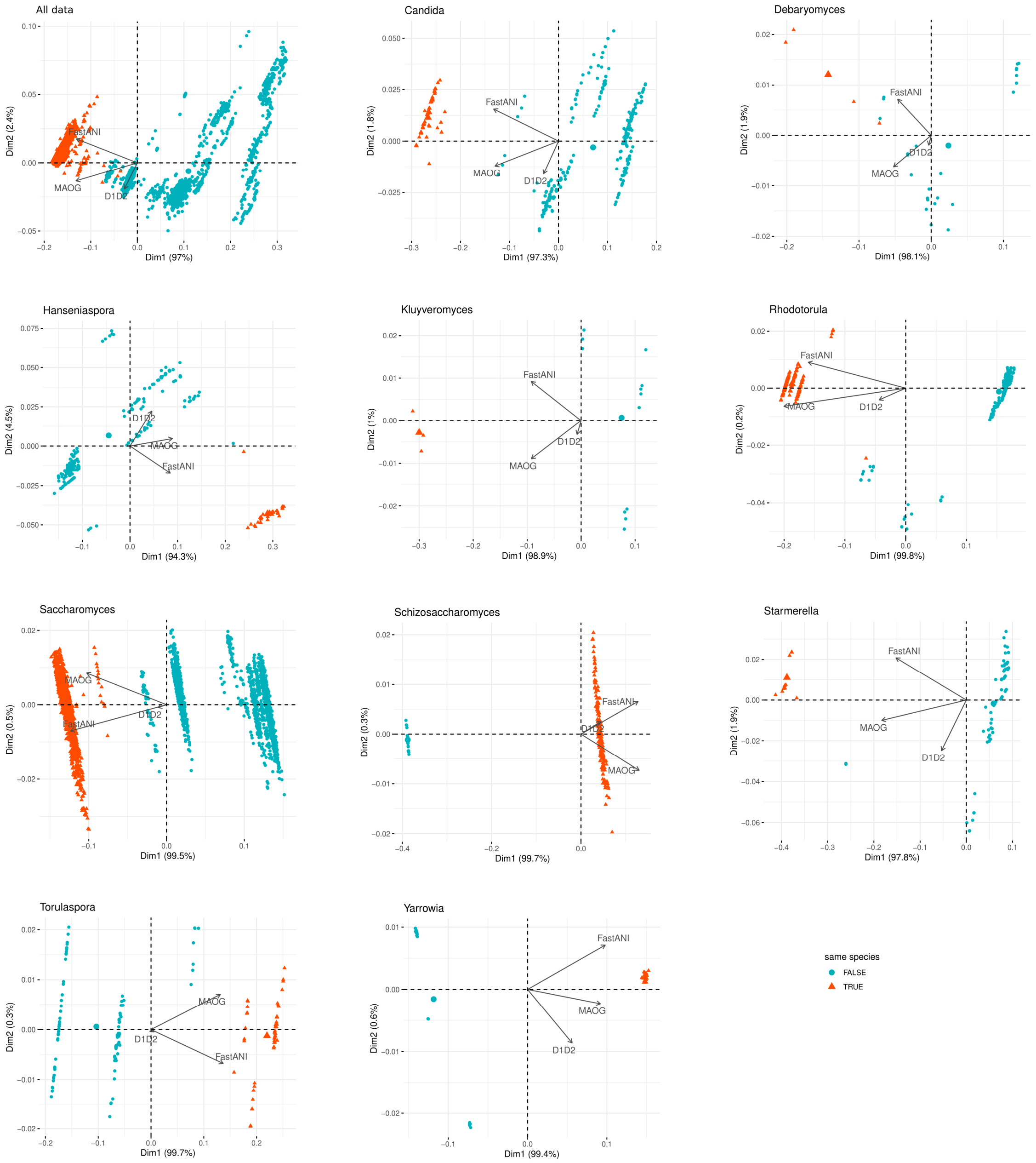

Figure S3. Principal component analysis (PCA) was performed to evaluate the capability of D1/D2 alignment, MAOG and FastANI to delineate species.
